# Uptake and intra-inclusion accumulation of exogenous immunoglobulin by *Chlamydia*-infected cells

**DOI:** 10.1186/1471-2180-8-213

**Published:** 2008-12-05

**Authors:** David V Pollack, Nancy L Croteau, Elizabeth S Stuart

**Affiliations:** 1 Department of Microbiology, University of Massachusetts, Amherst, Massachusetts, 01002, USA; 2 University of Massachusetts Medical School, Worcester Massachusetts, 01655, USA

## Abstract

**Background:**

Obligate intracellular pathogens belonging to the *Chlamydiaceae *family possess a number of mechanisms by which to manipulate the host cell and surrounding environment. Such capabilities include the inhibition of apoptosis, down-regulation of major histocompatability complex (MHC) and CD1/d gene expression, and the acquisition of host-synthesized nutrients. It is also documented that a limited number of host-derived macromolecules such as β-catenin and sphingomyelin accumulate within the inclusion.

**Results:**

This report provides evidence that immunoglobulin, inherently present in the extracellular environment *in vivo *and *in vitro*, enters infected cells and accumulates within the chlamydial inclusion. Using epi-fluorescent and confocal microscopy, this selective uptake of Ig is shown to occur among human leukocytes *in vivo *as well as cells cultured *in vitro*. These findings were confirmed by detection of IgG in the lysate of infected cells by western blot hybridization. Sequestered antibodies appear to be present during the entire course of the chlamydial developmental cycle and are distributed throughout this compartment. IgG pre-labeled with fluorescein, when added to the supernatant of infected cell cultures, was also imported and readily visualized. Accumulation of these molecules within the inclusion and the failure of bovine serum albumin or F(ab')_2 _fragments to accumulate in a similar manner suggests the process of entry is specific for intact IgG molecules and not a result of pinocytosis, diffusion, or any other mass endocytic event.

**Conclusion:**

Sequestration of a host cell-derived protein within the chlamydial inclusion, although unexpected, is not an unprecedented occurrence. However, selective accumulation of an exogenous host protein, such as extracellular IgG, has not been previously reported in connection with chlamydial infections. The selectivity of this process may indicate that this uptake plays an important role in pathogen physiology or virulence during infection and the phenomenon itself may give rise to novel diagnostic and therapeutic approaches.

## Background

*Chlamydia trachomatis *and *Chlamydophila pneumoniae *are ubiquitous, obligately intracellular human pathogens associated with ocular and respiratory tract infections, respectively. *C. trachomatis *is also the leading cause of bacterial sexually transmitted disease and represents the most commonly reported infectious agent in the United States, estimated to account for over $2 billion in domestic health care costs annually[1]. Additionally, strengthening links between these organisms and chronic diseases such as atherosclerosis, reactive arthritis, late-onset Alzheimer's, and asthma make advances in the understanding and treatment of these infections crucial for improved public health [2-5].

Members of the *Chlamydiaceae *family occupy and modify a membrane-bound vacuole termed an inclusion that has traditionally been believed to minimize their interaction with immune defenses and other host-derived molecules. As a result, this would reduce exposure to bacteriocidal factors and provide favorable conditions for chlamydial development. While perceived as privileged, a limited number of host macromolecules are known to be contained within the chlamydial inclusion, examples being β-catenin, sphingomyelin, CD63, and intermediate filament proteins [6-10]. It is also clear that several proteins associated with key aspects of infection, such as pathogen entry, do not localize within the inclusion; caveolin-1 and 2 are two such examples[11,12]. Similarly, many host peptides, including several Rab GTPases, associate with the inclusion membrane but do not enter the compartment[13]. Although the trafficking mechanisms involved for most of these inclusion-associated host proteins remain to be fully characterized, it is clear that the scarcity of intra-inclusion host proteins and the exclusion of key molecules suggest that the contents of this compartment accumulate selectively. Further evaluation of this privileged site, the types of host proteins within it, and the mechanisms by which they were sequestered will enhance understanding of chlamydial infections and may give rise to new methods for drug delivery and identification of infected cells.

## Results

### *In vitro *Detection of intracellular immunoglobulin within *Chlamydia*-infected cells

IgG sequestration within the chlamydial inclusion was first examined using cultured cell lines, conducted using J774A.1 murine macrophages infected with *C. trachomatis *serovar K. These *in vitro *experiments are based on the intra-inclusion sequestration of bovine IgG, a component of fetal bovine serum (FBS) that is routinely used as a supplement to tissue culture medium. The data yielded from these studies have indicated that extracellular IgG becomes co-localized with the chlamydial inclusion during infection.

**Figure 1:**
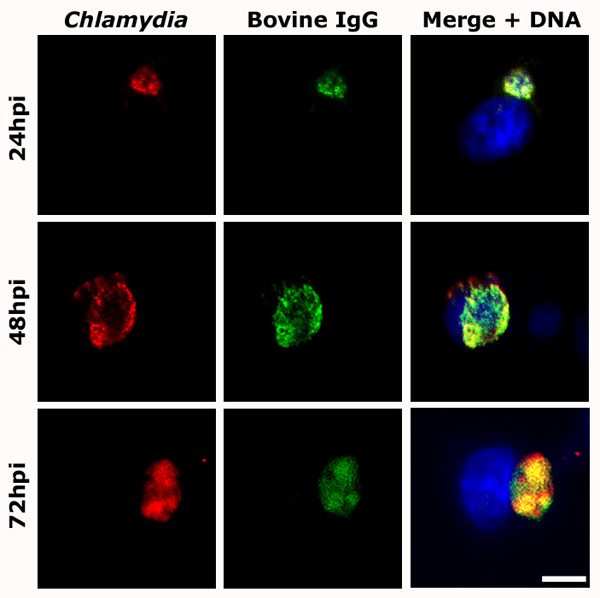
**IgG is present throughout the course of infection**. Selected time points throughout the course of a 72 h *C. trachomatis *serovar K infection in J774A.1 macrophages each show IgG to be co-localized with the chlamydial inclusion. FITC-labeled anti-bovine IgG antibodies (green) heavily co-localize with TRITC-labeled chlamydial antigens (red) at all observed timepoints, suggesting IgG is present during various stages of the chlamydial developmental cycle. A typical uninfected cell, detected by DAPI staining, is evident in the merge panel of the 48 hpi series and shows no antigen-specific recognition by the rabbit anti-*Chlamydia *or goat anti-bovine IgG antibodies. (bar = 15 μm).

In initial experiments, infected J774A.1 cells were fixed and stained for detection of *Chlamydia *and bovine IgG. As shown in Figure 1, coverslips immunostained at 24, 48, and 72 hours post infection (hpi) demonstrate that IgG is present within inclusions during both early and late stages of the developmental cycle. In contrast, uninfected cells in the same cultures (as observable at 48 hpi) show little to no evidence of intracellular bovine IgG. Cells stained only for *Chlamydia *failed to fluoresce in the FITC-channel, ruling out overlap of emission spectra as the cause of apparent IgG translocation (Additional File 1). The uptake of IgG has also been observed in cells infected by *C. trachomatis *serovar L2 and *C. pneumoniae*, the latter providing initial evidence that this occurs among both genera of *Chlamydiaceae *(data not shown). Although the exact cause and effect of such accumulation is unclear, internalization of IgG from the microenvironment surrounding infected cells could impact the efficacy of the host humoral response and/or play a role in chlamydial physiology.

It is important to note that visualization of nucleic acid with 4',6-diamidino-2-phenylindole (DAPI) confirms that the nuclei of uninfected and infected host cells are intact. Their healthy appearance indicates that the uptake of exogenous IgG by infected cells is not occurring due to porous cell membranes, breakdown of other cellular components, or as a function of impending cell lysis or death. The low level of IgG in neighboring uninfected cells and the observation that sequestered IgG is always associated with the presence of inclusions support the theory that these accumulations are a result of chlamydial infection.

### Co-localized IgG is contained within the chlamydial inclusion

**Figure 2:**
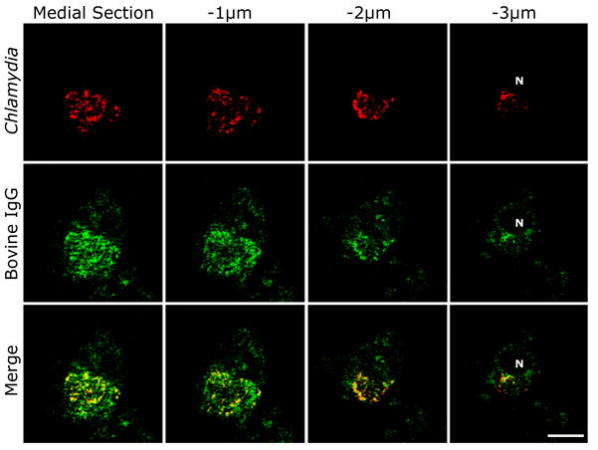
**Bovine IgG is sequestered within the chlamydial inclusion**. Merged images from a cross-sectional Z-series of a representative J774A.1 macrophage infected with *C. trachomatis *shows IgG distributed throughout the entire inclusion. Starting from the medial portion of the cell and sectioning downwards in 1 μm increments, co-localization (yellow) of chlamydial antigens and bovine IgG is observed throughout the thickness of the inclusion, indicating that IgG is contained within this compartment. For orientation, an "N" demarcates the nucleus that has been cross sectioned in the lowermost slice. Chlamydial organisms were labeled with a polyclonal rabbit anti-*Chlamydia *antibody and detected with TRITC-conjugated goat anti-rabbit IgG. IgG was labeled using a FITC-conjugated F(ab')_2 _goat anti-bovine IgG. (bar = 10 μm, section thickness = 0.5 μm)

Initial observations of IgG co-localization with the inclusion by epi-fluorescent microscopy led to evaluation of this phenomenon by optical sectioning of J774A.1 monolayers infected with *C. trachomatis *serovar K. A representative cross-sectional Z-axis series at 48 hpi was obtained using confocal microscopy and examined for internalized IgG (Fig. 2). These sections confirm that accumulated IgG localizes inside the chlamydial inclusion. Having provided verification that IgG is within the inclusion, the cross-sectioning of this and other cells excludes the possibility that nonspecific surface binding or autofluorescence led to the co-localization observed in the preceding experiments. Analysis of the inclusion using Z-axis sectioning also indicates that IgG is distributed throughout this entire compartment. When images of immune-labeled IgG and *Chlamydia *are merged (yellow), this distribution is apparent due to the presence of FITC-labeled IgG staining at the same locations as chlamydial antigens in all cross-sections.

### Confirmation of IgG sequestration by additional methods

**Figure 3:**
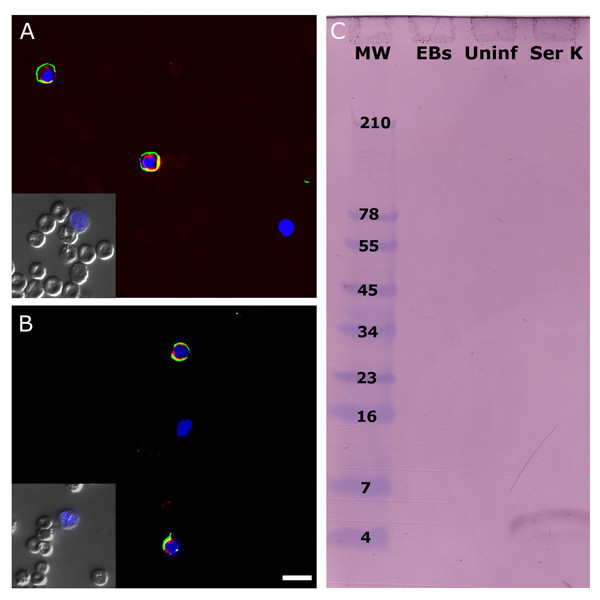
**Confirmation of IgG accumulation by additional methods**. Blood smears from two human donors, both with laboratory-confirmed chlamydial infections, were immunostained for *Chlamydia *(TRITC, red) and human IgG (FITC, green). Microscopy fields A and B contain three unidentified leukocytes each, two of which are infected and representative of all infected white cells observed *in vivo*. Probing with a monoclonal mouse anti-human IgG antibody (detected using FITC-conjugated goat anti-mouse IgG) demonstrates that detectable levels of human IgG are present in infected leukocytes. Yellow coloration represents instances of co-localization between IgG and chlamydial inclusions. Inclusions were labeled with a rabbit anti-*Chlamydia *polyclonal antibody followed by TRITC-conjugated goat anti-rabbit IgG. Nomarski DIC imaging on a portion of each field demonstrates the membrane integrity of both erythrocytes and infected leukocytes (insets) (bar = 20 μm). (C) Western blot hybridization analysis probing for bovine IgG was performed on renografin-purified *C. trachomatis *EBs (EBs) and lysates from uninfected (Uninf.) and infected cultures (Ser K). Cell lysates were matched to ensure equal numbers of cells per lane (~2.0 × 10^7 ^cells/mL) and purified EBs were diluted to the same IFU/mL as the infected the cell lysate (~1 × 10^6^IFU/mL).

The possibility that intra-inclusion IgG sequestration observed during *in vitro *infection was an artifact of the culture system led to confirmatory experiments using alternative methods. Human leukocytes capable of supporting chlamydial infection were evaluated after preparing smears of donor blood samples. Using methods similar to those applied to cell culture monolayers, these were immunostained for chlamydial antigens and human IgG. Microscopic examination of the samples indicated that chlamydial antigens and IgG were exclusively co-localized. Figures 3A and 3B consist of blood smears from two different donors, each containing three leukocytes per field. Immunolabeled human IgG (FITC, green) is detectably co-localized with anti-*Chlamydia *staining (TRITC, red). Nomarski DIC imaging (insets) and nuclear (DAPI, blue) staining indicate that the blood smears are well-preserved (also see additional 2). Thus, fragile erythrocytes in the same field that are often subject to rupture, remain intact. An example of an infected leukocyte is also included with each Nomarski DIC image and confirms that the membranes of these cells are also intact. The ability for a number of these cell types to become infected *in vivo *was previously demonstrated and quantified but no reports have indicated that there is differential susceptibility[14]. This suggests that *Chlamydia *may be capable of infecting IgG-producing plasma cells, which would result in the apparent co-localization of human IgG within the inclusion. However, studies evaluating the peripheral blood of healthy patients in comparison to those with multiple myeloma have indicated that normal donors do not have detectable circulating plasma cells, which are primarily located in lymph nodes[15].

Micrographs indicating that extracellular IgG is sequestered within *Chlamydia*-infected cells were corroborated by examining their IgG content using western blot hybridization (Fig. 3C). To compare levels of IgG contained within matched cultures of uninfected and infected cells, flasks were treated with trypsin to lift cells and remove surface-associated IgG. After thorough washing and lysis, these preparations were analyzed and provided validation for the immunofluorescent microscopy that had been performed. The polyclonal anti-bovine IgG antibody used to probe cell lysates detected high levels of intracellular IgG in infected cell lysates but failed to detect the same product in uninfected ones of matched cell number. This was evident through the appearance of an IgG-positive band at ~4 kDa in infected cell lysates, an apparent product of IgG breakdown, while uninfected J774A.1 cells contain a level of intracellular IgG that is undetectable.

To ensure that cross-reactive binding of the anti-bovine IgG antibody is not occurring during western blot analysis, purified *C. trachomatis *elementary bodies were probed simultaneously. The absence of any IgG-positive band in this lane indicates that the antibody has no reactivity with chlamydial organisms themselves.

### Sequestration of extracellular IgG is selective

Based on the observations described, the potential selectivity and specificity of protein uptake into the chlamydial inclusion by infected cells was also evaluated. Fetal bovine serum (FBS), the source of IgG sequestered within the inclusion of cells *in vitro*, also contains the osmoregulatory protein bovine serum albumin (BSA). At ~66 kDa, this protein is less than half the molecular weight of IgG and present in ≥ 50-fold higher concentration, constituting more than 50% of the total protein in FBS. Because of its abundance and low molecular weight relative to IgG, infected cells were immuno-probed for BSA as a control designed to assess the selectivity of the phenomenon.

**Figure 4:**
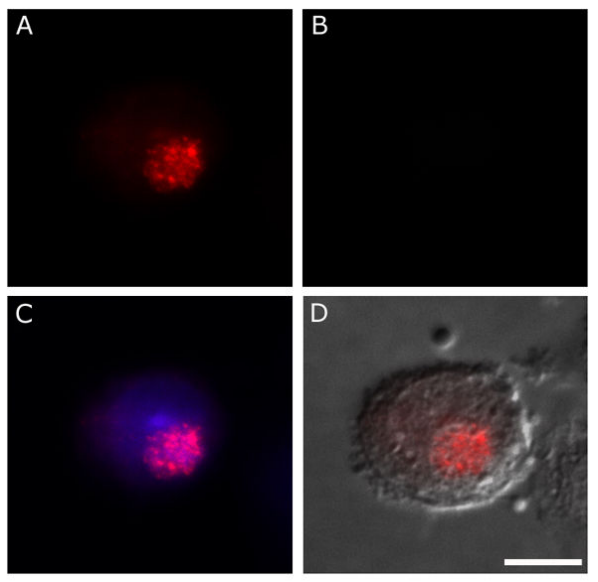
**Bovine serum albumin does not accumulate within infected cells**. Cells infected for 48 h with *C. trachomatis *serovar K (guinea pig anti-*Chlamydia*/TRITC-conjugated goat anti-guinea pig)(A) were probed for internalized BSA using a rabbit anti-BSA antibody and detected with FITC-conjugated goat anti-rabbit Ig (B). Unlike IgG, this abundant ~66 kDa polypeptide does not accumulate within the chlamydial inclusion, as apparent in the merge of *Chlamydia *and nuclear stains (C). A merge incorporating DIC imaging shows an identifiable inclusion and indicates no co-localization of the two immunolabels has occurred (D). This demonstrates that BSA is not subject to the same entry or sequestration as bovine IgG, indicative that the process of IgG internalization is selective. (bar = 10 μm).

Monolayers of J774A.1 cells infected with *C. trachomatis *serovar K were incubated as in previous experiments and probed to identify chlamydial antigens and BSA. Epi-fluorescent microscopy indicates that BSA does not accumulate within the chlamydial inclusion as observed with IgG (Fig. 4). The exclusion of BSA is significant because, should IgG uptake occur by nonselective mechanisms such as pinocytosis, diffusion, or phagocytosis, BSA would be susceptible to the same phenomenon. Therefore, the failure of BSA to enter infected cells supports the conclusion that IgG sequestration is a selective process, rather than a result of mass, non-specific endocytosis. Biological systems with analogous specificities typically achieve them by means of a receptor-mediated process and research in this area is in progress.

### Fluorescently-labeled antibodies are subject to sequestration

**Figure 5:**
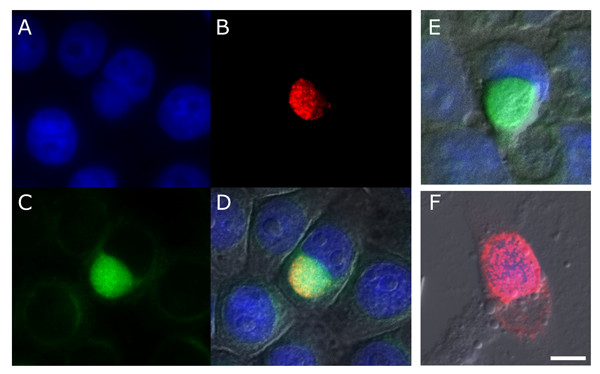
**Sequestration of pre-labeled IgG within the chlamydial inclusion**. Cells infected with *C. trachomatis *serovar K were incubated at 24 hpi with FITC-labeled IgG and fixed 1 h later. Visualization of DNA using DAPI (A) confirms that the J774A.1 macrophages are intact and robust. Rabbit anti-*Chlamydia *antibodies labeled with TRITC-conjugated anti-rabbit IgG (B) shows a well-developed inclusion while accumulation of FITC-labeled IgG (C) within it is readily apparent. A merge of images A-C with a DIC micrograph (D) shows the cells are generally healthy during infection. (E) Cells that were similarly exposed to FITC-labeled IgG but were not permeabilized, show sequestration of the labeled IgG. In this absence of anti-*Chlamydia *staining DIC imaging and DAPI were used to identify infected cells. (F) *Chlamydia*-infected cells were also incubated with FITC-labeled F(ab')_2 _fragments. These cells exhibit no intracellular accumulation of this fluorescent label. (bar = 10 μm).

Despite appropriate controls, the use of secondary antibodies for indirect staining can result in misleading, artifactual findings. In light of this concern, monitoring the uptake of a FITC-labeled IgG can eliminate the risk of cross-reactivity and non-specific binding by the secondary antibody, both inherent with the indirect technique. The cells were only exposed to FITC-labeled IgG in their growth medium prior to fixation then, after extensive rinsing as described in the methods, monolayers were permeabilized and stained for *Chlamydia *with a TRITC-conjugated secondary antibody. Importantly, at no point after the cells were permeabilized did they come in contact with FITC-labeled IgG. Epi-fluorescent microscopy of J774A.1 cells with intact nuclei (Fig. 5A), when infected with *C. trachomatis *serovar K (Fig. 5B), exhibit intra-inclusion accumulation of the FITC-labeled IgG (Fig. 5C) following its addition to the culture media. These labeled molecules are present in detectable but minimal levels throughout the cytosol of uninfected cells shown in the same field. Nomarski DIC imaging indicates that both uninfected and infected cells appear otherwise healthy and possess intact plasma membranes (Fig. 5D). Monitoring the uptake of FITC- labeled molecules effectively addresses concerns regarding antibody promiscuity and other artifacts of post-fixation manipulation. In addition, this technique provides further validation of the experiments presented above that show bovine IgG uptake and accumulation within the inclusion. The same experiment was also performed without indirect immunolabeling of *Chlamydia *or cell membrane permeabilization. Instead, the inclusion was identified using DAPI labeling and Nomarski imaging after fixation with paraformadehyde, avoiding permeabilization of cell membranes. These control measures confirm that green fluorescence detected within the inclusion pictured in Fig. 5E is not an artifact of staining and results from the uptake of FITC-labeled IgG.

An additional control experiment was designed to assess whether translocation requires a structurally complete IgG molecule. FITC-labeled F(ab')_2 _fragments were similarly added to the supernatant of infected cultures. Unlike intact IgG molecules, these F(ab')_2 _fragments failed to enter the inclusion as evident in Figure 5F. The exclusion of F(ab')_2 _molecules from the inclusion suggests the Fc region of the IgG molecule, which these fragments lack, is necessary for the observed sequestration. These data, in addition to results obtained when probing for intracellular BSA, indicate the observed IgG uptake is not a result of porous membranes, structural damage, or mass transport.

### Verification of IgG sequestration during infection of non-phagocytic cell lines

**Figure 6:**
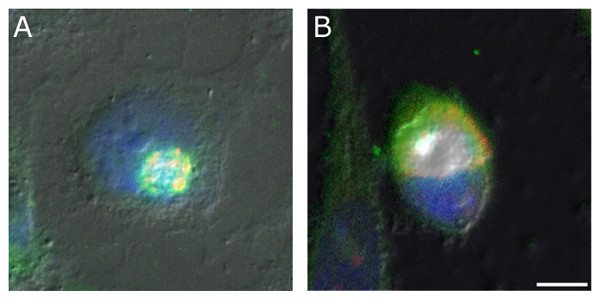
**IgG sequestration occurs in multiple cell lines**. Non-phagocytic cells infected for 48 hpi were evaluated for the presence of bovine IgG within the chlamydial inclusion. Human epidermal HEp-2 (A), and mouse fibroblast McCoy (B) cell lines were evaluated by epi-fluorescence (1000×) and both were found to exhibit co-localization (yellow) of bovine IgG (detected with FITC-conjugated goat anti-bovine IgG, green) and the chlamydial inclusion (rabbit anti-*Chlamydia*/TRITC-conjugated goat anti-rabbit IgG, red), thus indicating that IgG has been translocated to the inclusion in these cell lines as well. (bar = 10 μm).

J774A.1 murine macrophages have been used for the majority of IgG uptake characterization, their phagocytic nature making them useful cells for chlamydial research due to their susceptibility to infection. However, as shown in Figure 6, IgG sequestration within the inclusion also occurs in other established cell lines such as HEp-2 and McCoy.

The human epidermoid carcinoma cell line HEp-2, which is commonly used in chlamydial and viral research, was evaluated for the presence of IgG within the inclusions of infected cells. Epi-fluorescent microscopy again indicates that exogenous bovine IgG is co-localized with the inclusions of infected HEp-2 cells (Fig. 6A) and the distribution of staining was indistinguishable from that of J774A.1 cells. A second immortalized cell line, the McCoy cell, has traditionally been utilized for *in vitro *cultivation of *C. trachomatis*[16]. This mouse fibroblast line is readily infected and hardy throughout the course of chlamydial development. These cells were similarly infected with *C. trachomatis *serovar K and, as with the other cell lines evaluated, epi-fluorescence microscopy indicated the presence of IgG within the inclusion (Fig. 6B). Although not shown, all cell types were also evaluated with confocal microscopy and the observed IgG sequestration was confirmed in all instances. Taken together, results from the examination of different cell lines suggest that this phenomenon could affect a variety of cell and tissue types within the infected host, including cells of the endothelium, epithelium, and blood.

## Discussion

Historically the chlamydial inclusion has been viewed as an isolated, inaccessible vesicle within the host cell. Many host-pathogen interactions remain undefined and only a limited number of host-derived molecules are known to be co-opted by chlamydial organisms and stored within this compartment. In addition, the function and consequences of their sequestration remain unclear in some cases. Possible effects of sequestering these molecules include immune evasion, fulfillment of physiological requirements, or facilitating enhanced inclusion development. Further questions arise because molecules known to accumulate within the inclusion share little in common with one another. These include host polypeptides involved in cell-cell adhesion and cytoskeletal structures, cell membrane components, and eukaryotic phospholipids[17]. Examples of these diverse molecules include β-catenin, sphingomyelin, intermediate filament proteins, and cholesterol[7,8,10,18]. The limited number but diverse nature of macromolecules that are sequestered within the inclusion implies that the removal of these host components may also have an impact on cell or tissue function, resulting in sequelae that appear unrelated to infection.

With the exception of exogenous cholesterol, the intra-inclusion sequestration of moieties derived from the extracellular environment of the host cell is yet to be reported[18]. The experiments described have provided evidence that extracellular IgG is among the proteins now known to be sequestered within the inclusion. To our knowledge, application of FITC-labeled IgG to the culture media has allowed for the first successful demonstration of non-invasive, direct targeting of developing inclusions with an exogenous molecule, a capability that could give rise to novel therapeutics. The data presented suggest the uptake of IgG to be a selective, directed phenomenon as opposed to a non-discriminatory influx of molecules resulting from phagocytic or pinocytic events. It is also clear that this process occurs throughout the normal course of infection, a factor that could indicate its importance to chlamydial development. Previously unreported with other intracellular pathogens, these features make IgG uptake and accumulation a unique aspect of chlamydial infections and one requiring further characterization. A more thorough understanding of this phenomenon will result in unprecedented access to this privileged compartment, including innovative methods of detection and treatment. A macrolide-IgG conjugate, for instance, would theoretically result in an accumulation of bacteriocidal compound inside the pathogen-containing compartment[19].

Entry and intracellular accumulation of exogenous proteins can be a result of various mechanisms. Phagocytosis, fluid-phase endocytosis, and internalization during chlamydial entry are non-specific mass transport processes that might lead to the events observed. If this were the case then it would also lead to a detectable influx of BSA into the cell. However, the absence of the smaller, more abundant BSA from the inclusion rules out involvement of non-specific mechanisms for IgG uptake. These findings suggest that entry of IgG is a selective process, possibly a receptor-mediated phenomenon.

The specificity of this sequestration is further emphasized by results obtained using directly-labeled fluorescent molecules to track the uptake of IgG. The accumulation of FITC-labeled IgG and exclusion of FITC-labeled F(ab')_2 _fragments indicate that all or part of the IgG Fc region is necessary for the observed translocation of the molecule and excludes Ig association with EBs as the key mechanism for entry. These findings support a role for host cell Fc receptors in the mechanism of IgG entry and, along with the exogenous nature of IgG, distinguishes this phenomenon from other examples of host cell-derived protein translocation to the chlamydial inclusion. The experiments using fluorescently-labeled whole IgG molecules indicate that accessing and labeling of the inclusion without fixation or permeabilization may be possible.

IgG internalization within the inclusion is also clearly not restricted to phagocytic cells. As professional phagocytes, it is reasonable that J774A.1 cells would be more likely to acquire IgG from the extracellular environment, possibly leading to its sequestration within the inclusion. However, the results obtained during tests for the same phenomenon in HEp-2 and McCoy cells demonstrate that IgG uptake and accumulation occurs with non-phagocytic cells as well, and with seemingly indistinguishable efficiencies. It is also observed that this occurs with all infected cells in these cultures, making intra-inclusion IgG sequestration a ubiquitous phenomenon in the cell lines tested. The demonstration that this can occur in diverse cell types *in vitro *suggests it could play an important role in a variety of tissues during chlamydial infection in a human host. Given the various of cell types shown to exhibit IgG uptake during infection, this could potentially impact Ig levels in the interstitial space surrounding infected cells *in vivo*, ultimately compromising host immune defenses at sites of infection.

Western blot hybridization data has demonstrated that IgG breakdown products are present at high concentrations in infected cells, supporting the theory that this protein is being degraded. As large, globular proteins that are widely distributed throughout most mammalian tissues, Ig molecules would represent an abundant source of amino acids, should breakdown and subsequent assimilation by *Chlamydia *occur. This is not without precedent among agents of infectious disease. A number of bacterial pathogens, including species of *Neisseria*, *Haemophilus*, and *Streptococcus*, produce well-characterized proteases catalyzing the hydrolysis of human IgA, an important virulence factor during infection[20,21]. It is also known that several bacterial species contain IgG proteases[22,23]. In addition, at least one eukaryotic organism, the pork tapeworm *Taenia solium*, is capable of converting these immune molecules to a supplemental nutrient source through the use of an IgG protease [24]. Given the existence of homologous bacterial proteases and a eukaryotic system in which the pathogen is known to exploit IgG for its own metabolic needs, the uptake and degradation of IgG by *Chlamydia *to enhance nutrient availability would not be unique to this organism. Further, should antibodies be found to constitute a substrate of chlamydial physiology, it would provide a possible rationale for the increased non-specific, polyclonal antibody production by B cells in response to the presence of chlamydial organisms, as demonstrated in early work by Bard and Levitt[25]. For these reasons, a thorough evaluation of whether Ig breakdown products are being used for chlamydial metabolism will be necessary and may provide additional insight into chlamydial physiology and host cell interaction.

It is clear that the translocation reported here is distinct from the previously described examples of host protein uptake into the inclusion and further studies will be required to define the mechanisms of initiation and to assess the fate of sequestered IgG. This phenomenon has not been previously reported with any intracellular pathogen, suggesting that Ig uptake and accumulation may be a unique characteristic of *Chlamydia*. Additionally, under experimental conditions, this phenomenon has shown promise as a means of targeting the inclusion in a direct and specific manner. This would provide a valuable new tool for studies of *Chlamydia *both *in vivo *and *in vitro*, relevant because of the pathogen's prevalence and significant impact on public health. A more complete understanding of host Ig sequestration should provide new insights regarding the detection, evaluation, and treatment of chlamydial infections.

## Conclusion

Results obtained from these studies demonstrate intra-inclusion IgG sequestration as a newly observed characteristic of *Chlamydia*-infected cells. Evidence from these studies support the follow conclusions: i) IgG, initially present only in the media surrounding the host cell, is translocated to the inclusion of infected cells but shows minimal internalization by uninfected cells, consistent with the basal level of uptake that is involved in Ig homeostasis[26]. ii) The localization of IgG is within the inclusion compartment itself. iii) The observed translocation of IgG is specific for this particular extracellular protein. iv) Whole IgG is necessary for uptake since antibodies pre-labeled with fluorescein are subject to the sequestration while pre-labeled F(ab')_2 _fragments are not. v) IgG sequestration occurs in a variety of cell types and is therefore not unique to phagocytic cells such as the J774A.1 line examined initially. Under the conditions tested, significant accumulation of IgG is dependant on the presence of *C. trachomatis *or *C. pneumoniae *organisms in the host cell. These *in vitro *microscopy studies were validated with the detection of IgG sequestration in human blood donor samples and by western blot of infected cultures. Together, the findings introduce a unique characteristic of cells infected with *Chlamydia*, both *in vitro *and *in vivo*, and might also have implications for pathogenesis and treatment of these diseases.

It is too early to conclude the precise mechanism(s) leading to sequestration of IgG within the chlamydial inclusion. Although it is likely that this phenomenon is the result of combined bacterial and host cell processes, the molecular and cellular function of this pathogen-host interaction remains to be clarified. This report, while possibly indicative of host cell Fc receptor involvement in IgG uptake, is only intended to describe the previously unreported presence of exogenous IgG within the chlamydial inclusion. Ongoing studies are designed to further characterize the mechanisms driving this sequestration from the perspective of both the eukaryotic host cell and the pathogen. Given its occurrence *in vivo *and the potential applications for drug delivery, detection, and genetic manipulation of chlamydial organisms, future examinations of IgG sequestration will likely provide important tools and valuable knowledge in the context of chlamydial infections and their treatment.

## Methods

### Cell Culture

Prior to inoculation, cells were grown in Richter's Improved Minimal Essential Medium with Insulin (IMEMZO)(Irvine Scientific, Santa Ana, CA) supplemented with 10% FBS (Atlanta Biologicals, Norcross, GA) at 37°C and 5% CO_2_. J774A.1, HEp-2, and McCoy cells were seeded on 12 mm glass coverslips and grown until ~80% confluent for infection, staining, and visualization.

### Infection of host cells

For all infections, whole cell lysate in sucrose phosphate glutamate buffer (SPG) of J774A.1 cells infected with *C. trachomatis *serovar K/UW-31Cx was diluted 1:100 from a stock inoculum in Cycloheximide Overlay Medium (COM) (Cambrex Bio Science, Walkersville, MD) supplemented with 10% FBS. This inoculum was incubated with cells for 3 h at 37°C, removed, and replaced with fresh COM/FBS for further incubation. Cell monolayers, with the exception of those used for Fig. 5E, were fixed and permeablized for 10 minutes with 70% cold MeOH to facilitate immunostaining.

### Immunostaining

After removing MeOH, slides and coverslips were washed 3× with PBS prior to staining. All antibodies were diluted in PBS (pH 7.2) then placed in 300 μL and 500 μL aliquots on coverslips and slides, respectively. Incubations were performed in the dark for 1 h at room temperature. Detection of chlamydial organisms infecting J774A.1, HEp-2, and McCoy cells was performed using a rabbit anti-*C. trachomatis *polyclonal antibody (Biodesign, Saco, ME) diluted 1:100. Binding of anti-*Chlamydia *antibodies was detected with a tetramethyl-rhodamine isothiocyanate (TRITC)-conjugated goat anti-rabbit IgG (H+L) secondary antibody (Jackson ImmunoResearch, South Windham, ME) diluted 1:100. Additional confirmatory immunostaining was obtained by using a mouse monoclonal anti-chlamydial LPS antibody (BioDesign, clone BDI815) followed by a TRITC-conjugated goat anti-mouse IgG (H+L) (Jackson ImmunoResearch, South Windham, ME), both diluted 1:100. For detection of sequestered bovine IgG in J774A.1, HEp-2, and McCoy cells a FITC-conjugated F(ab')_2 _fragment anti-bovine IgG (H+L) (Jackson ImmunoResearch, South Windham, ME) was used at a 1:100 dilution. BSA labeling on permeabilized, infected J774A.1 cells was performed using a 1:200 dilution of rabbit anti-bovine albumin polyclonal antibody (Pel-Freez Biologicals, Rogers, AR) followed by a 1:100 dilution of FITC-labeled goat anti-rabbit IgG polyclonal antibody (Jackson ImmunoResearch, South Windham, ME). *Chlamydia *staining during the BSA experiment was performed using pre-diluted guinea pig anti-*Chlamydia *antibody (Biomeda Corp., Hayward CA) followed by a 1:100 dilution of TRITC-conjugated goat anti-guinea pig IgG (Jackson ImmunoResearch, South Windham, ME). All coverslips and slides were mounted with VectaShield DAPI-containing mounting media (Vector Laboratories Inc., Burlingame, CA).

### Detection of sequestered IgG in human leukocytes

A 30 μL aliquot of a fraction from whole blood enriched for leukocytes from two individuals confirmed to be infected with *Chlamydia sp*. and collected during a separate large-scale study of human donor blood[14], was smeared on a glass slide and stained for evaluation of IgG sequestration *in vivo*. The sample was preserved in ethylenediaminetetraacetic acid (EDTA) and was fixed and permeabilized with 70% cold MeOH. Labeling of the inclusion was achieved using the using the same staining protocol described for *in vitro *experiments. Human IgG was immunostained using a mouse monoclonal antibody specific for the human Fc fragment of IgG (Pel-Freez Biologicals, Rogers, AR), diluted 1:1000, followed by a 1:100 dilution of FITC-conjugated goat anti-mouse antibody (Jackson ImmunoResearch, South Windham, ME), both for 1 h at RT.

### Demonstration of uptake using labeled IgG

IgG translocation and accumulation inside the inclusions of infected cells was tracked using commercially-prepared, pre-labeled immunoglobulin. At 24 hpi a 1:100 dilution of FITC-conjugated goat anti-human IgG (H+L) or FITC-conjugated F(ab')_2 _fragment rabbit anti-horse IgG (H+L) was added to the media of infected cells, which consisted of COM supplemented with 10% FBS. Coverslips were fixed 1 h later with 70% cold MeOH then stained for DNA and *Chlamydia *as described. Control cells were fixed with 0.1% paraformaldehyde after media removal and thorough rinsing, then mounted with DAPI-containing VectaShield.

### Confirmation of intracellular IgG by western blot hybridization

A 75 cm^2 ^culture flask of J774A.1 cells infected with *C. trachomatis *serovar K and one mock infected flask, all at 80% confluence, were treated with 0.25% trypsin from porcine pancreas (Sigma-Aldrich Inc., St. Louis, MO) until cells had lifted, centrifuged at 550 × g for 5 minutes, and resuspended in 1 mL of PBS (pH 7.2) per treatment (~2 × 10^7^/mL cells for each treatment). Infected and uninfected cell preparations were lysed and treated with deoxyribonuclease I (Sigma-Aldrich Inc., St. Louis, MO) and ribonuclease A (Sigma-Aldrich Inc.), both used at 50 μg/mL for 4 h at 37°C. Purified EBs were diluted to match the IFU of infected cell lysate (~1 × 10^6 ^IFU/mL). After mixing with 2× non-reducing sample buffer (50% ddH_2_O, 12.5% 0.5 M tris-HCl pH 6.8, 10% glycerol, 2.5% bromophenol blue, 2.0% SDS), 20 μL of each preparation was electrophoresed using a 10–20% Tricine SDS-PAGE (Invitrogen Corp., Carlsbad, CA) and transferred to Immobilon-P 0.45 μm PVDF membrane (Millipore Corp., Bedford, MA) at 80 V for 1 h. Membrane was blocked with nonfat dry milk, 6% in ddH_2_0, for 3 h, rinsed 3× with PBS, and probed with an alkaline phosphatase-conjugated rabbit anti-bovine IgG polyclonal antibody (Jackson ImmunoResearch, South Windham, ME) at a 1:1000 dilution for 1 h at RT. After washing 3× with PBS the membrane was developed with Sigma Fast 5-bromo-4-chloro-3-indolyl phosphate/nitroblue tetrazolium (BCIP/NBT) tablets (Invitrogen Corp., Carlsbad, CA) for 15 min before washing with dH_2_O and allowing to dry.

### Microscopic analysis

Confocal microscopy on adherent monolayers and smeared blood samples was performed using an LSM 510 Meta Confocal Microscope at 630× magnification (Carl Zeiss AG, Oberkochen, Germany). The Z-series presented in Figure 2 contains optical sections, acquired in sequential 1.0 μm increments with a thickness of 0.5 μm, throughout the Z-axis of the cells. Epi-fluorescent microscopy was performed at 400× or 1000× using a Nikon Spot epi-fluorescent microscope (Nikon, Tokyo, Japan) with Spot-RT camera system (Diagnostic Imaging). Both instruments were used for Differential Interference Contrast (DIC) imaging.

## Authors' contributions

DVP conducted experiments on J774A.1 and HEp-2 cells, performed analysis using confocal and epi-fluorescent microscopy, western blotting, and drafted this manuscript. NLC first observed the phenomenon described, confirmed its specificity for Ig by probing for BSA, and evaluated *in vivo *samples. ESS supervised all research, was instrumental in experimental design, and co-wrote the final manuscript with DVP. This research was carried out as thesis work for a M.S. degree (NLC) and partial fulfillment of the requirements for a Ph.D. degree (DVP) in the department of Microbiology at the University of Massachusetts, Amherst.

## Supplementary Material

Additional File 1 **Indirect immunostaining control.** In addition to using indirect immunolabeling of cultured cells for both *Chlamydia *and IgG in Figures 1, 2, and 6, controls were stained with only the anti-*Chlamydia *antibody. Epi-fluorescent microscopy shows no green FITC signal to be detectable when DAPI, FITC, and TRITC channels are merged. This demonstrates that IgG apparent within the inclusion is not the result of anti-*Chlamydia *antibody cross-reactivity. Additionally, this excludes spectral overlap of TRITC detectable in the FITC emission spectrum as the cause of the reported findings. Click here for file

Additional File 2 **Staining controls on *in vivo *samples.** Blood samples from human donors known to be infected with *Chlamydia *were assessed for IgG accumulation within leukocytes. Staining controls were performed in order to assess the cross-reactivity and spectral overlap of the antibodies used. Blood from the same samples shown in Figure 3 was also stained only for *Chlamydia *(A) or IgG (B). Given the absence of any green coloration, merged images of the detected signals in DAPI, FITC, and TRITC emission ranges indicate that spectral overlap into the FITC channel is not occurring. Click here for file
