# Supplementary figures and images for: Uptake and intra-inclusion accumulation of exogenous immunoglobulin by Chlamydia-infected cells
Source: BMC Microbiol. 2008 Dec 5;8:213. doi: 10.1186/1471-2180-8-213 (PMC2621372; doi:10.1186/1471-2180-8-213)

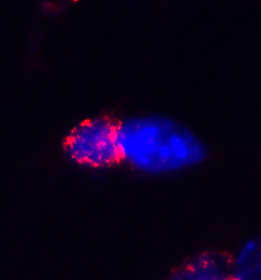

Supplement: Additional File 1 — Indirect immunostaining control. In addition to using indirect immunolabeling of cultured cells for both Chlamydia and IgG in Figures 1, 2, and 6, controls were stained with only the anti-Chlamydia antibody. Epi-fluorescent microscopy shows no green FITC signal to be detectable when DAPI, FITC, and TRITC channels are merged. This demonstrates that IgG apparent within the inclusion is not the result of anti-Chlamydia antibody cross-reactivity. Additionally, this excludes spectral overlap of TRITC detectable in the FITC emission spectrum as the cause of the reported findings. [file 1471-2180-8-213-S1.jpeg]

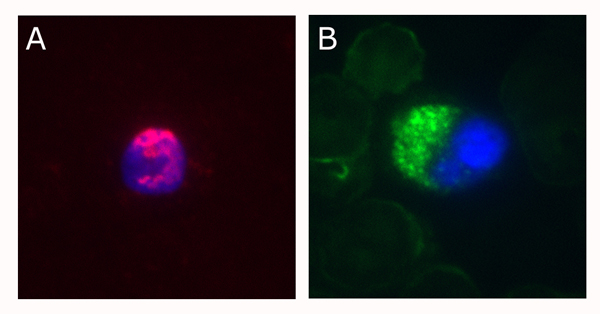

Supplement: Additional File 2 — Staining controls on in vivo samples. Blood samples from human donors known to be infected with Chlamydia were assessed for IgG accumulation within leukocytes. Staining controls were performed in order to assess the cross-reactivity and spectral overlap of the antibodies used. Blood from the same samples shown in Figure 3 was also stained only for Chlamydia (A) or IgG (B). Given the absence of any green coloration, merged images of the detected signals in DAPI, FITC, and TRITC emission ranges indicate that spectral overlap into the FITC channel is not occurring. [file 1471-2180-8-213-S2.jpeg]
